# Supplementary material for: High density lithium niobate photonic integrated circuits
Source: Nat Commun. 2023 Aug 10;14:4856. doi: 10.1038/s41467-023-40502-8 (PMC10415301; doi:10.1038/s41467-023-40502-8)
Supplement: Supplementary file 1 — Supplementary Information [file 41467_2023_40502_MOESM1_ESM.pdf]

Supplementary Information to: High density lithium niobate photonic integrated circuits

Zihan Li,[1,2*] Rui Ning Wang,[1,2*] Grigory Lihachev,[1,2*] Junyin Zhang,[1,2] Zelin Tan,[1,2] Mikhail Churaev,[1,2] Nikolai Kuznetsov,[1,2] Anat Siddharth,[1,2] Mohammad J. Bereyhi,[1,2,3] Johann Riemensberger,[1,2] Tobias J. Kippenberg[1,2†]

[1]*Institute of Physics, Swiss Federal Institute of Technology Lausanne (EPFL), CH-1015 Lausanne, Switzerland*

[2]*Center of Quantum Science and Engineering (EPFL), CH-1015 Lausanne, Switzerland*

[3]*Luxtelligence SA, CH-1015 Lausanne, Switzerland*

[†]*tobias.kippenberg@epfl.ch*

[*] *These authors contributed equally to this work.*

Diamond-like Carbon Etch mask

We deposit diamond-like carbon film on the lithium niobate on insulator (LNOI) wafer in a Oxford Plasmalab 80 refitted with a CH_4 precursor gas dosing line. The deposition parameters and cleaning cycles are optimized to avoid particle contamination on the films. To etch the DLC film with oxygen plasma, we deposit a thin Si_3N_4 film on the DLC film a hard mask for DLC etching (see in Supplementary Fig. 1(a)). After DUV stepper photolithography, we transfer the pattern from the $400\,\mathrm{nm}$ M108Y photoresist layer (see in Supplementary Fig.1(b)) to the Si_3N_4 film with a fluoride-based reactive ion etching process (see in Supplementary Fig. 1(c)). Then the photonic circuit pattern is transferred to DLC film with an O_2 plasma etch step (see in Supplementary Fig. 1(d)) and the etching of the $LiNbO_3$ film is performed using Ar^+ ion beam.

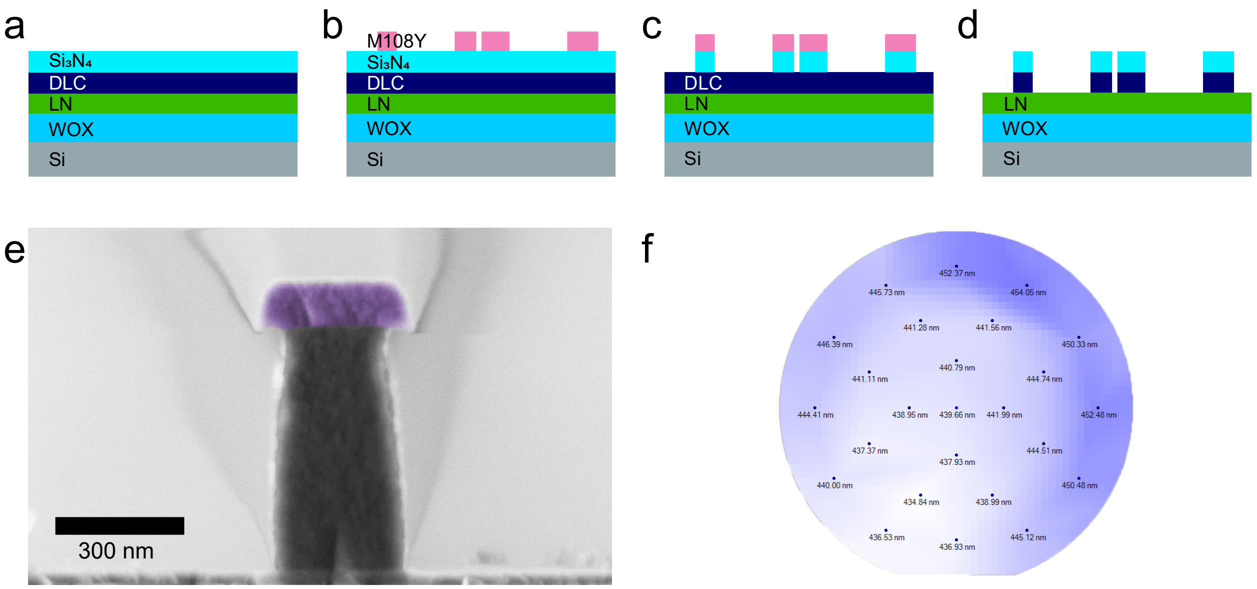

Figure 1: Process flow for DLC mask etching. **(a)** Deposit DLC and Si_3N_4 film on the commercial LNOI wafer. **(b)** Create the pattern layer with DUV stepper photolithography. **(c)** Etch the Si_3N_4 film with a DUV photoresist mask and fluorine plasma. **(d)** Etch the DLC film with Si_3N_4 mask. The photoresist is also etched in the same step. **(e)** The SEM image for the DLC mask cross-section of a taper. The Si_3N_4 is colored in purple and DLC in black. **(f)** Wafer-map of thickness distribution measurements of PECVD-deposited DLC film.

$LiNbO_3$ etching with different methods

We compared the different dry etching methods for $LiNbO_3$ at first. With fluorine and chlorine chemistry plasma etching, we observe a rough surface due to the redeposition of non-volatile etch products LiF or LiCl (see Supplementary Fig. 2(a,b)). Hence, we selected the purely physical argon bombardment as the etching method. Using only vertical impingement plasma beams, we observe a slanted sidewall with a large number of redeposition products and distinctive trenching around the waveguide base (see Supplementary Fig. 2(c)). The observed trenching is related to the reflection of argon ions from the slanted sidewalls. By optimizing the angle between the argon ions and the wafer surface, we can reduce the redeposition of volatile etch products, and remove trenching by ion reflection (see Supplementary Fig. 2(d)), as well as increase the sidewall angle of the optical waveguide.

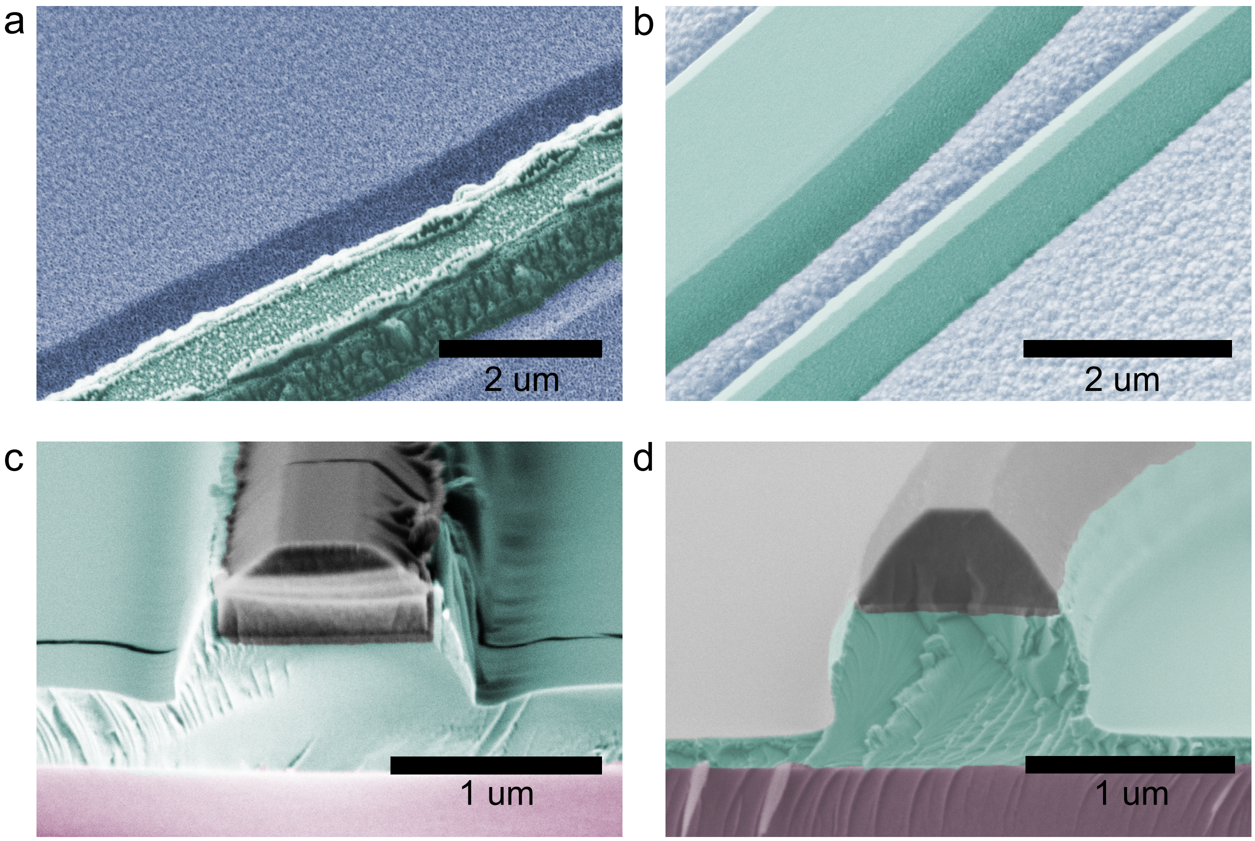

Figure 2: Comparison of different dry etching methods for $LiNbO_3$waveguides. The $LiNbO_3$waveguide etched by fluorine **(a)** and chlorine **(b)** plasma with DLC mask. The DLC mask is stripped after the etching. The cross-section of the $LiNbO_3$waveguide etched by argon plasma or ion in RIE **(c)** and IBE **(d)**. The $LiNbO_3$waveguide is colored in green blue, $LiNbO_3$slab in blue, SiO_2in light purple and DLC in black.

Electro-optic efficiency comparison for strip and ridge waveguides

We compare the electro-optic efficiency of two approaches to integrated lithium niobate photonics: First, the strip waveguide approach discussed in the manuscript and second, the ridge waveguide approach representative of state of the art in $LiNbO_3$-based integrated photonics [1,2]. We calculate the main metric of electro-optic modulation – the half-wave voltage length product, which describes the required voltage needed for the optical phase shift of π along a $1\,\mathrm{cm}$ electro-optic

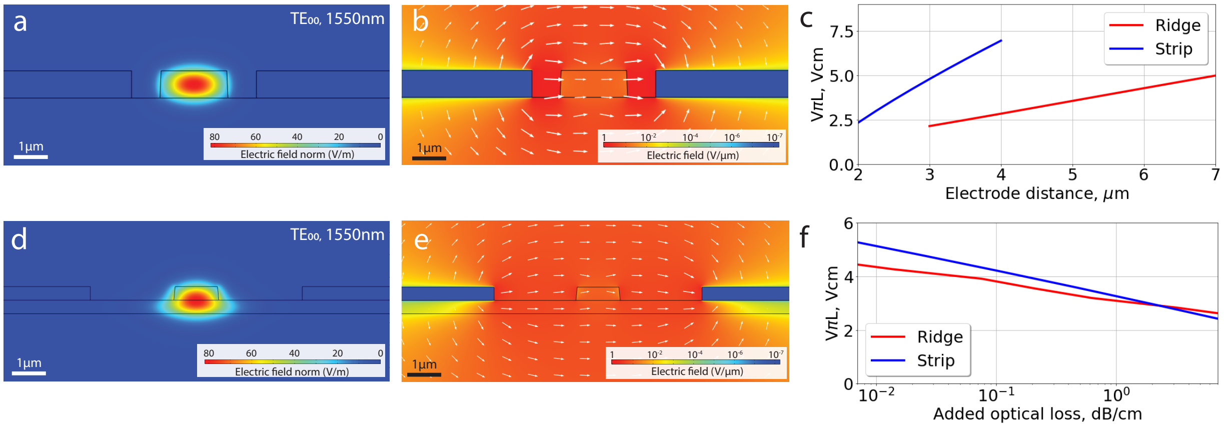

Figure 3: Comparison of half-wave-voltage for strip and ridge $LiNbO_3$waveguides. (a) FEM simulation of optical field distribution in strip and ridge **(d)** waveguides. **(b)** FEM simulation of electric field distribution in strip and ridge **(e)** waveguides with $3.5\,\mu m$ and $3.5\,\mu m$ electrodes distance. **(c)** Simulated modulator half-wave-voltage vs electrode distance for strip (blue) and ridge (red) waveguides. **(f)** Comparison of electro-optic efficiency as a function of added optical loss due to the presence of conductive electrodes.

waveguides:

$$V_\pi L = \frac{\lambda}{2n^3 r_{33} E_{\mathrm{eff}}} \cdot 1\mathrm{V},$$

where $E_{\mathrm{eff}} = \frac{\int_{\mathrm{LN}} |E_{x,\mathrm{opt}}|^2 E_{x,\mathrm{el}} dS}{\int |E_{x,\mathrm{opt}}|^2 dS}$ is the effective electric field that takes into account the overlap integral between modulation electric field and the optical field distribution, r_{33} is $LiNbO_3$electro-optic coefficient, λ is optical wavelength, and n is the optical effective index [3]. We consider X-cut $LiNbO_3$ wafer and TE-polarization of optical mode, such that the principal axis of the optical and electric fields are parallel to the z-axis of $LiNbO_3$ crystal. We performed FEM simulations of the optical waveguide mode distribution (see Supplementary Fig. 3(a,d)) and the electrostatic field distribution between the metal electrodes corresponding to a set of distances between electrodes(see Supplementary Fig. 3(b,e)) using COMSOL Multiphysics. In particular, we compare a strip waveguide with $2\,\mu m$ width and $0.6\,\mu m$ height and a ridge waveguide with $1.5\,\mu m$ width and $0.35\,\mu m$ slab thickness. The ridge waveguide shows better performance (lower Vπ values) at the same electrode distance (see Supplementary Fig. 3(c)) due to the presence of $LiNbO_3$ slab, that "guides" the electric field into the optical mode. In the case of strip waveguide, the modulation

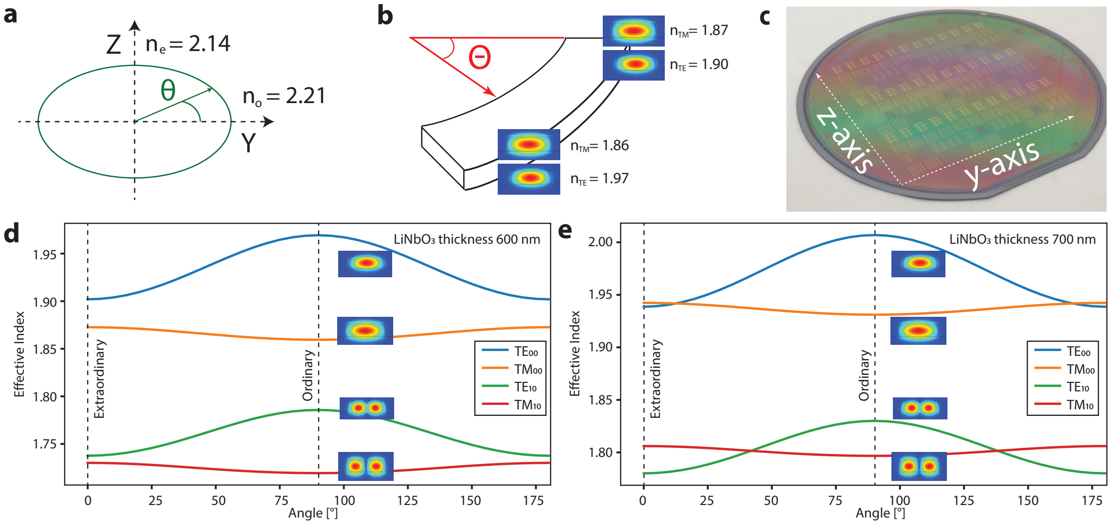

Figure 4: Birefringent mode mixing in sub-micron waveguides (a) Definition of crystal axes in the simulation. **(b)** Illustration of fundamental TE and TM modes and their respective refractive indices across the waveguide bends. **(c)** Illustration of the $LiNbO_3$crystal axes across a wafer. **(d)** Numerical simulation of effective mode indices for the fundamental and first higher order modes for both polarization directions along a waveguide bend as a function of the relative angle of the propagation direction and the crystal Y-axis for $600\,\mathrm{nm}$ thick strip waveguides. **(e)** Same as (d) but for $700\,\mathrm{nm}$ thick strip waveguides.

electric field is accumulated mainly in the air gap between the electrodes and the waveguide because of the large dielectric constant ($\epsilon \approx 28$) of the $LiNbO_3$ extraordinary crystal axis. On the other side, the tight optical confinement of strip waveguides makes it possible to place electrodes closer to the waveguide, conserving the same added optical loss as for the ridge waveguide. We included optical mode dissipation at the surface of the conductive electrodes to correctly compare the performance of the two platforms based on the numerical calculations of the dielectric function of gold by Werner et al. [4]. As depicted in Fig.3, the strip waveguide has a decrease of less than 20% in voltage length product across the full range of low-loss regimes. At moderate ohmic losses of 0.1 dB/cm, the performance penalty is around 10% and at ohmic losses in excess of 1 dB/cm, the strip waveguide outperforms the ridge waveguide. Here we consider both cases to be air-cladded as was discussed in the main text. The dielectric cladding between electrodes and waveguide can

significantly improve electro-optic performance by mitigating the electric field screening effect but might lead to higher optical losses due to the increased evanescent optical field.

Birefringence induced optical mode mixing in waveguide bends

On the ring resonator dispersion measurements, we observe strong mode splitting induced by the mode mixing between different mode families (see Figure 2 main text and Supplementary Figure 4). We identify the mode mixing behaviour as the intermixing of fundamental TE and TM modes by comparison of the group indices of both modes. In contrast to Si_3N_4 and SI-based optical waveguides, we observe this particular mode mixing behaviour for both microring resonators and racetrack resonators with optimized waveguide bends [5,6]. We explain this behaviour of strip and ridge waveguides by the phase matching of TE and TM modes due to the material birefringence of the negative uniaxial $LiNbO_3$ crystal. In any waveguide bend, the optical mode experiences a change of the material index as the projection of its principal axes onto the crystal axes rotates (see Fig. 4(a,b,c)). The in-plane TE mode experiences a much more significant change of material refractive index as its major electric field direction aligns with the extraordinary crystal axes for horizontal waveguides and the ordinary crystal axes for vertical waveguides. Our numerical simulations indicate that the TE and TM modes experience phase matching at specific waveguide direction angles relative to the crystal extraordinary axis (see Fig.4(d,e)). This phase matching induces mode mixing that leads to distortion of the dispersion profile (see Fig. 5(a)). Further simulations reveal that the phase matching of the two modes occurs in any waveguide thicker than $700\,\mathrm{nm}$ irrespective of the etching depth, sidewall angles, or slab heights and for both SiO_2cladded and uncladded waveguides.

Hybrid integrated laser characterization

Supplementary Figure 5 shows the frequency-dependent transmission, cavity linewidth and histogram of intrinsic microresonator loss rate of the $LiNbO_3$ chips used for hybrid integrated laser demonstration in Figures 3 and 4 of the main manuscript (D101 F2 C4 WG204). We use the funda-

mental TE mode in all cases for characterization and laser operation. We employ frequency comb calibrated laser spectroscopy to perform linear characterization of photonic chips [7].

Mach-Zehnder modulator

We fabricate a MZM modulator with a 1.73 cm length (see Supplementary Fig. 7) in x-cut $LiNbO_3$with push-pull configuration that has waveguide width of 1.5 μm and an $LiNbO_3$waveguide – electrode gap of 2 μm. $LiNbO_3$waveguides are formed by partial etching with a 100 nm slab thickness and the electrodes are fabricated in gold with 500 nm thickness. We use MMI splitters with less than 100 μm length that provide less than 5% imbalance in splitting. Measured V_π is 1.94 V (see Supplementary Fig. 7(c)), resulting in $V_\pi L$=3.3 V$\times$cm.

Comparison of lithium niobate platforms

Table 1 presents a comparison of different demonstrations lithium niobate platforms in terms of of fabrication method, intrinsic Q-factor, linear optical loss, VπL product, EO tuning efficiency.

Ref.	Fabrication	Intrinsic Q-factors	Linear optical loss	Statistical analysis	$V_\pi L$ product, EO efficiency
This work	DUV stepper+DLC	$\approx 10^7$	0.04 dB/cm	yes	3.35 V·cm
[1]	EBL	$\approx 10^7$	0.03 dB/cm	no	no data
[8]	EBL	1.2×10^6	0.3 dB/cm	no	≈ 300 MHz·V^{-1}
[9]	EBL	2.5×10^6	no data	no	500 MHz·V^{-1}
[10]	EBL	7.8×10^6	0.06 dB/cm	no	1.6 V·cm
[11]	DUV stepper	1.8×10^6	0.27 dB/cm	yes	no data
[12]	Laser writing	7.1×10^6	no data	no	2.16 V·cm
[13]	EBL	no data	no data	no	2.5 V·cm
[14]	EBL	no data	no data	no	2.15 V·cm
[15]	EBL	no data	0.41 dB/cm	no	no data
[16]	EBL	4×10^6	no data	no	no data
[17]	EBL	1×10^6	no data	no	no data

Table 1: Performance comparison of integrated lithium-niobate-based platforms.

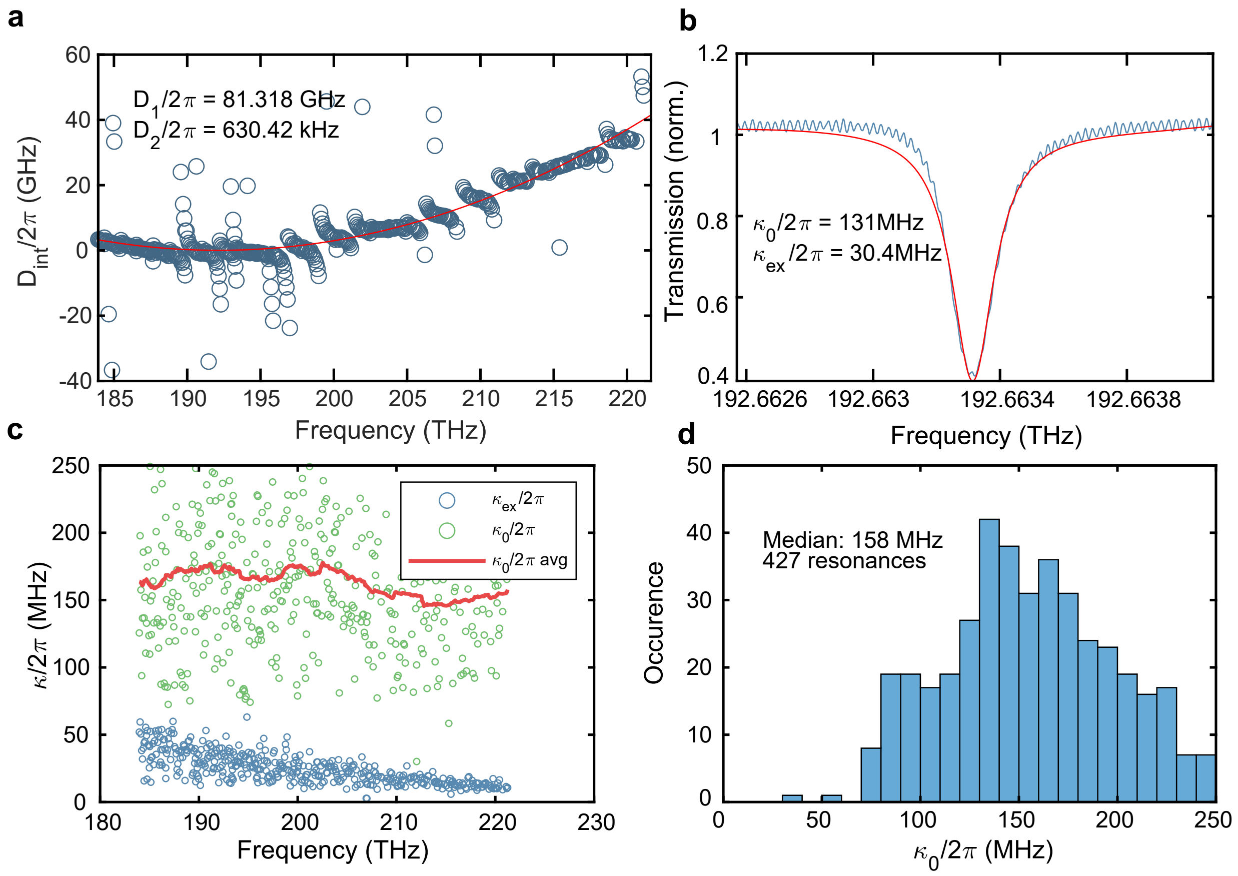

Figure 5: **(a)** Integrated dispersion of LNOI microresonator with free-spectral range (FSR) $81.318\,\mathrm{GHz}$ and anomalous GVD of $630\,\mathrm{kHz}$. **(b)** Normalized transmission of the racetrack microcavity resonance at $192.663\,\mathrm{THz}$ with Lorentzian fit resulting in $131\,\mathrm{MHz}$ intrinsic cavity loss and $30.4\,\mathrm{MHz}$ bus waveguide coupling rate. **(c)** Frequency-dependent microresonator loss $\kappa_0/2\pi$ (green), bus waveguide coupling $\kappa_{ex}/2\pi$ (blue) and moving average of intrinsic loss (red). **(d)** Histogram of intrinsic microresonator loss rate measured for 427 resonances with the median value of $158\,\mathrm{MHz}$.

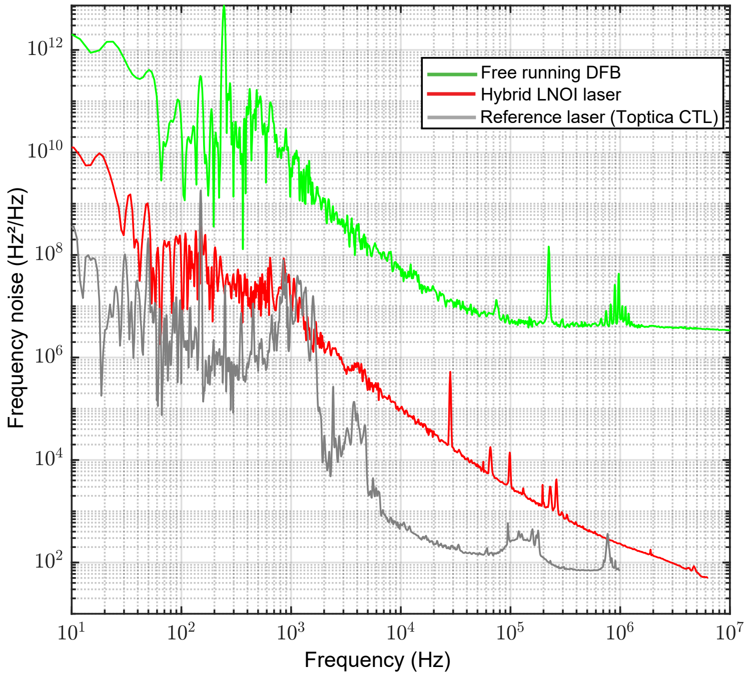

Figure 6: Single-sided power spectral density of the hybrid LNOI laser frequency noise self-injection locked to the racetrack resonator featuring $15\,\mathrm{GHz}$ FSR (red), in free-running regime (green). The frequency noise of the reference laser is presented in grey.

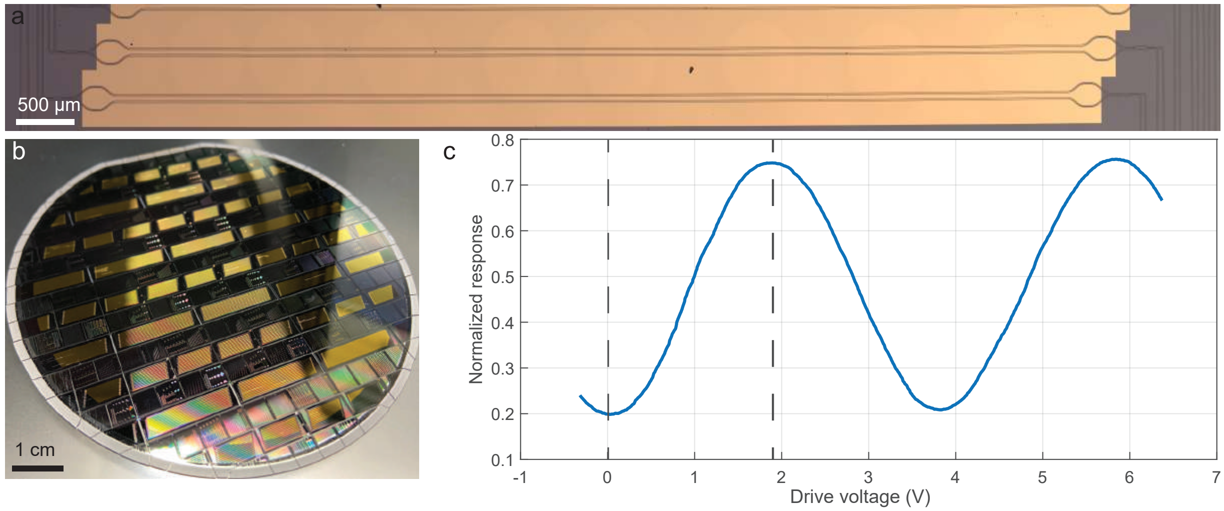

Figure 7: **(a)** Optical image of 1.73 cm long MZM modulators. **(b)** Photo of the 100 mm x-cut LN wafer with MZMs. **(c)** V_π measurement for a MZM modulator with 1.73 cm length resulting in V_π=1.94 V.

Supplementary References

1. Zhang, M., Wang, C., Cheng, R., Shams-Ansari, A. & Lončar, M. Monolithic ultra-high-Q lithium niobate microring resonator. *Optica* **4**, 1536–1537 (2017).

2. He, M. *et al.* High-performance hybrid silicon and lithium niobate mach–zehnder modulators for 100 Gbit s^{-1} and beyond. *Nature Photonics* **13**, 359–364 (2019).

3. James E. Toney. *Lithium Niobate Photonics* (Artech House Publishers, 2015).

4. Werner, W. S., Glantschnig, K. & Ambrosch-Draxl, C. Optical constants and inelastic electron-scattering data for 17 elemental metals. *Journal of Physical and Chemical Reference Data* **38**, 1013–1092 (2009).

5. Ji, X. *et al.* Compact, spatial-mode-interaction-free, ultralow-loss, nonlinear photonic integrated circuits. *Communications Physics* **5**, 1–9 (2022).

6. Ye, Z. *et al.* Integrated, ultra-compact high-Q silicon nitride microresonators for low-repetition-rate soliton microcombs. *Laser & Photonics Reviews* **16**, 2100147 (2022).

7. Del'Haye, P., Arcizet, O., Gorodetsky, M. L., Holzwarth, R. & Kippenberg, T. J. Frequency comb assisted diode laser spectroscopy for measurement of microcavity dispersion. *Nature Photonics* **3**, 529–533 (2009).

8. Li, M. *et al.* Integrated pockels laser. *Nature Communications* **13**, 5344 (2022).

9. Zhang, M. *et al.* Broadband electro-optic frequency comb generation in a lithium niobate microring resonator. *Nature* **568**, 373–377 (2019).

10. Desiatov, B., Shams-Ansari, A., Zhang, M., Wang, C. & Lončar, M. Ultra-low-loss integrated visible photonics using thin-film lithium niobate. *Optica* **6**, 380–384 (2019).

11. Luke, K. *et al.* Wafer-scale low-loss lithium niobate photonic integrated circuits. *Opt. Express* **28**, 24452–24458 (2020).

12. Fang, Z. *et al.* Efficient electro-optical tuning of an optical frequency microcomb on a monolithically integrated high-q lithium niobate microdisk. *Opt. Lett.* **44**, 5953–5956 (2019).

13. Xu, M. *et al.* High-performance coherent optical modulators based on thin-film lithium niobate platform. *Nature Communications* **11**, 3911 (2020).

14. Shams-Ansari, A. *et al.* Electrically pumped laser transmitter integrated on thin-film lithium niobate. *Optica* **9**, 408–411 (2022).

15. Krasnokutska, I., Tambasco, J.-L. J., Li, X. & Peruzzo, A. Ultra-low loss photonic circuits in lithium niobate on insulator. *Optics express* **26**, 897–904 (2018).

16. He, Y. *et al.* Self-starting bi-chromatic linbo3 soliton microcomb. *Optica* **6**, 1138–1144 (2019).

17. Gong, Z., Liu, X., Xu, Y. & Tang, H. X. Near-octave lithium niobate soliton microcomb. *Optica* **7**, 1275–1278 (2020).
